# Feasibility and Early Experience with Pediatric Open Access Endoscopy: A Pilot Study

**DOI:** 10.3390/pediatric17060134

**Published:** 2025-12-17

**Authors:** Monique T. Barakat, Dorsey M. Bass, Roberto Gugig

**Affiliations:** 1 Divisions of Adult and Pediatric Gastroenterology & Hepatology, Stanford University School of Medicine, Palo Alto, CA 94305, USA; 2 Division of Pediatric Gastroenterology, Stanford University Medical Center, Palo Alto, CA 94305, USA

**Keywords:** endoscopy, pediatric, open access

## Abstract

Background: Open access endoscopy (OAE) allows outpatient endoscopic procedures without prior consultation with the endoscopist, a practice common in adult gastroenterology but not part of pediatric gastroenterology practice. Here we evaluate the feasibility and safety of a newly implemented pediatric OAE program. Methods: We identified patients aged 18 and under who underwent OAE in the first year of our program using a prospectively maintained endoscopy database. The program involved three experienced endoscopists and included demographics, indications, interventions, and adverse events. Patients/parents received follow-up calls on day 1 and day 7 to detect adverse events and assess perceptions of the OAE process. Results: A total of 54 outpatient OAE procedures were performed, with a median patient age of 10 years (range 18 months–18 years). This included 33 esophagogastroduodenoscopies (EGDs) and 16 colonoscopies, all with biopsies. ERCPs were performed for stone management (4) and stricture evaluation/stent exchange (1). All procedures were successful with no adverse events reported, and patient/parent feedback indicated that the OAE approach was beneficial in terms of lifestyle, socioeconomic, and psychological aspects. Some challenges were identified through follow-up discussions. Conclusions: Our early experience suggests that pediatric OAE is feasible and appeared safe within this small pilot cohort, with no adverse events observed. Advantages of pediatric OAE include minimizing missed school days and reducing medical anxiety. Feedback has led to refinements in practice at our institution, and further study on OAE is warranted at the endoscopy society level. Larger studies are needed to determine safety, effectiveness, and generalizability.

## 1. Introduction

Endoscopy is a basic element of diagnosis and management of gastrointestinal (GI) disorders for adults and children. Historically, the process of undergoing an endoscopic procedure involved a consultation with the gastroenterologist performing the procedure or his/her close colleague to allow for a comprehensive evaluation of the patient’s medical history, symptoms, and indications for the procedure. However, in the past two decades, Open Access Endoscopy (OAE), where endoscopy is performed without prior consultation with the gastroenterologist performing the procedure or a close colleague, has transformed this paradigm, particularly in adult gastroenterology, where it has gained significant traction [1]. In fact, the majority of adult endoscopists have integrated OAE into their practice, facilitating timely access to endoscopic interventions without the need for prior specialist consultation [2]. Reasons for this shift to open access endoscopy for adult patients include the fact that endoscopy has become more routine and innumerable studies attest to the safety of routine endoscopic procedures such as upper endoscopy and colonoscopy. Large studies of OAE in adult endoscopy practice have found this practice to be effective, safe and a viable mainstream approach for performing endoscopic procedures more expeditiously [3]. The diagnostic yield of open access endoscopy procedures has been found to be high, and comparable with the yield of standard access endoscopy, within the United States (US) and internationally [4]. Risk stratification for sedation modality has also been found to be effective for open access endoscopy procedures [5].

In the past several years, pediatric endoscopic procedures have become much more commonly performed, and the safety of these procedures has been confirmed and quality metrics defined, with the risk profiles well characterized [6,7]. This evolution of pediatric endoscopy prompted our consideration for a role for OAE in pediatric endoscopy. Despite the success of OAE in adult populations, its application in pediatrics has remained very limited and the historical practice of clinic consultation prior to endoscopy remains the norm at most centers. Pediatric endoscopy typically adheres to a more traditional model, where a gastroenterology consultation at the institution performing the endoscopy is the norm—even for patients referred by outside gastroenterologists. This approach is often attributed to the unique complexities associated with pediatric patients, including their developmental considerations, varying presentations of gastrointestinal disorders, and the need for tailored pre-procedural assessments. The only study evaluating OAE in pediatric patients prior to the present study was conducted over 10 years ago evaluated only satisfaction of the practice and found that patient pre-procedure mood and patient/parent satisfaction were lower for patients who underwent OAE [8]. Perhaps in part due to concerns about patient satisfaction, the majority of pediatric gastroenterology practices have not adopted OAE.

Delays in endoscopic care for children and adolescents, often characterized by a longer than ideal interval between referral and pre-endoscopy clinic consultation are a common challenge in our pediatric endoscopy program and, anecdotally, in many pediatric endoscopy programs. Recognizing the need for improved access to endoscopic services for pediatric patients, our institution initiated a pediatric OAE program in 2022. This innovative approach aims to streamline the referral process, allowing primary care providers and outside gastroenterologists to refer patients directly for outpatient endoscopic procedures without the prerequisite of a prior consultation. Through implementation this program, we sought to enhance the efficiency of care delivery, reduce wait times for procedures, and ultimately improve patient outcomes for the subset of patients who could benefit from OAE.

In this manuscript, we present data from our initial experience implementing this pediatric OAE program to analyze the feasibility, safety, and potential benefits of OAE for the first time in pediatric patients. Implementing OAE in pediatrics requires consideration of several factors that differ from adult practice, including developmental communication needs, caregiver involvement, institutional sedation policies, and consent/assent processes. These distinctions underscore the need to evaluate feasibility and safety specifically in pediatric settings.

## 2. Methods

### 2.1. Study Design and Population

This pilot study was conducted as a retrospective analysis using a prospectively maintained endoscopy database at our institution. We identified patients aged 18 years and under who underwent Open Access Endoscopy (OAE) during the first year of our OAE program. This study was conducted in accordance with the ethical standards of our institution and approved by the institutional review board (IRB).

### 2.2. OAE Program Implementation

The OAE program was initiated with the involvement of three experienced endoscopists who are experienced in performing endoscopic procedures. These endoscopists were selected based on their extensive experience and comfort level with outpatient endoscopic interventions.

Because this was a pilot implementation, referrals were screened by the participating endoscopists, and only patients with appropriate indications and minimal comorbidities were included. This selective process may limit generalizability but reflects real-world considerations for safe early adoption.

The OAE program was developed as a pathway to facilitate outpatient endoscopic procedures without prior consultation with a gastroenterologist or endoscopist. The program aimed to streamline the referral process for pediatric patients, allowing primary care providers and outside gastroenterologists to refer patients directly for endoscopic evaluation.

### 2.3. Data Collection

Data were collected from our endoscopy database, including demographic characteristics (age, sex, and medical history/comorbidities), indications for the procedures, interventions performed, and any adverse events that occurred during or following the procedures. Adverse events were defined as any complications or unexpected outcomes related to the endoscopic procedures.

### 2.4. Follow-Up Protocol

To enhance the detection of adverse events and assess patient/parent perceptions of the OAE process, follow-up calls were conducted by our clinical team on day 1 and day 7 post-procedure using a standardized telephone script. During these calls, patients or their parents were asked about any symptoms or complications experienced since the procedure, as well as their overall satisfaction with the OAE process. Satisfaction was rated on a 0 to 5 scale in addition to free text comments from families.

### 2.5. Qualitative Free Text Analysis

Parent, patient and endoscopist free text comments submitted via pre- and post-procedure open-ended telephone surveys were analyzed qualitatively using NVivo12TM software (QSR International, Doncaster, VIC, Australia). A language coding analysis was performed to identify thematic nodes supported by the content and sentiment of the comments. Additionally, we evaluated the frequency of words in the free text responses to create a word cloud, where the size of each word reflects its occurrence in the respondents’ comments. The word cloud was generated using wordcloud.com (accessed 8 January 2025). A thematic analysis was conducted to identify recurring concepts and perceptions. Word clouds were used as visual adjuncts but were not the primary analytic method. Themes were derived independently by two reviewers and reconciled by consensus.

### 2.6. Statistical Analysis

Descriptive statistics were employed to analyze the collected data. Frequencies and percentages were calculated for categorical variables, while means and standard deviations were computed for continuous variables where applicable. The analysis aimed to provide a comprehensive overview of the demographic characteristics, indications for procedures, interventions performed, and the incidence of adverse events associated with the OAE program.

## 3. Results

### 3.1. Patients and Procedures

**Figure 1:**
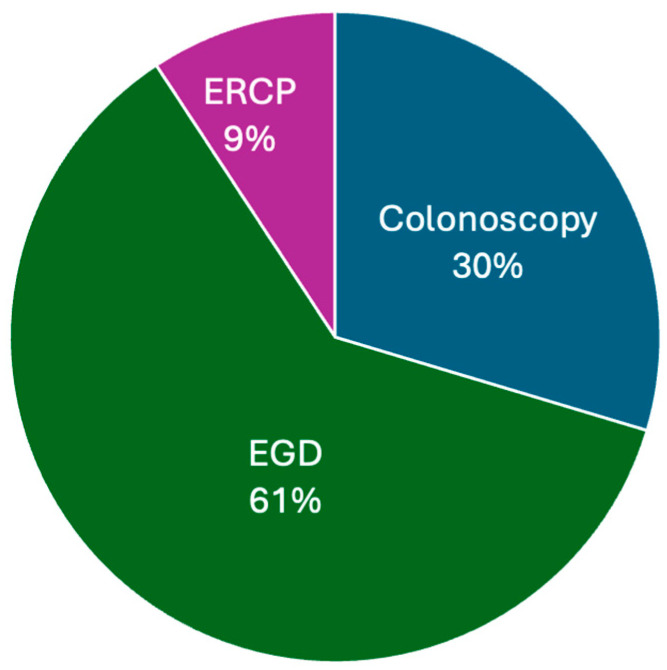
Distribution of OAE Procedures by Type. This bar graph illustrates the distribution of the different types of OAE procedures performed, including EGD, colonoscopy, and ERCP.

During the study period, a total of 54 outpatient Open Access Endoscopy (OAE) procedures were performed on pediatric patients, with a median age of 10 years (range: 18 months to 18 years). The distribution of procedures included 33 esophagogastroduodenoscopies (EGD), 16 colonoscopies, and 5 endoscopic retrograde cholangiopancreatographies (ERCP) (Figure 1). Inclusion criteria: All direct referrals for endoscopy were evaluated during the study period by one of the experienced endoscopists who had committed to performing OAE as part of this study. Indication for the procedure was assessed, as well as patient co-morbidities. If indication was appropriate from the perspective of the referring physician (all were for patients who were referred during this study period) and if patient comorbidities were deemed to be minimal or none (all but one patient screened met this criteria), and if caregiver/parent consented to OAE during scheduling (all parents/caregivers did during the study period), patients proceeded to undergo OAE. Exclusion criteria: Questionable indication for the procedure, significant comorbidities that could reasonable affect the risk profile of the procedure and/or lack of parental/caregiver consent for OAE were exclusion criteria for this OAE pilot study.

### 3.2. Procedure Details and Interventions

**Figure 2:**
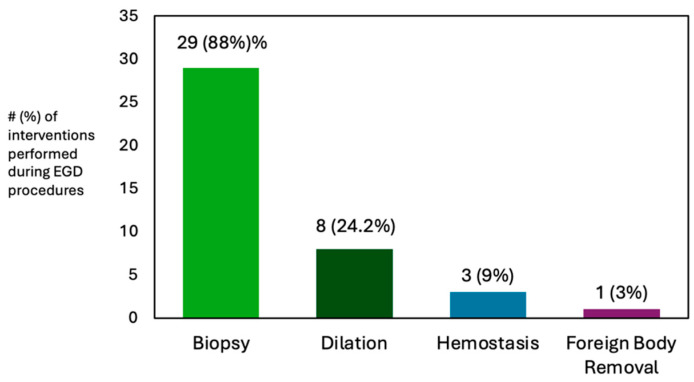
Esophagogastroduodenoscopy (EGD) interventions performed during open access endoscopy procedures.

Among the EGD procedures, the specific interventions included dilation (*n* = 8), hemostasis (*n* = 3), foreign body removal (*n* = 1), and biopsy (*n* = 29) (Figure 2). All 16 colonoscopy procedures involved biopsy. The majority of bowel preparations during colonoscopy were adequate for assessment and diagnosis (15/16, 93.8%) and one bowel preparation was fair (not ideal for assessment and diagnosis, but diagnostic biopsies were obtained and limited endoscopic evaluation was conducted (1/16, 6.3%). The terminal ileum was successfully reached and evaluated in all colonoscopy cases. The ERCP procedures were performed for stone management (*n* = 4) and stricture evaluation/stent exchange (*n* = 1).

### 3.3. Procedure-Associated Adverse Events

All OAE procedures were completed successfully, with no adverse events reported post-procedure for any of the procedures performed. To ensure adequate capture of procedure associated adverse events, follow-up telephone calls to assess patient and parent perceptions were conducted, per standard post-procedure assessment protocol and these calls did not result in capture of any additional procedure associated adverse events. Follow-up calls were successful in reaching all OAE patients in this study.

### 3.4. Qualitative Themes from Patient and Caregiver Feedback

**Figure 3:**
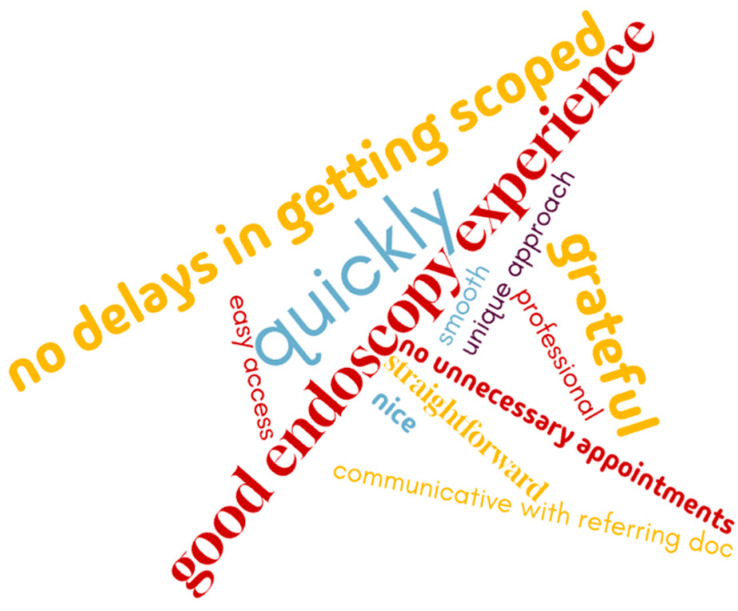
Word cloud depicting parent/patient perceptions reported via pre- and post-procedure open-ended survey of the open access endoscopy experience. Word cloud shown for illustrative purposes; themes derived from qualitative analysis are summarized in the text.

Analysis of all comments from patients, families and endoscopists regarding the OAE process revealed overwhelmingly positive feedback as assessed by the natural language processing analysis to detect ‘positive’ vs. ‘negative’ comments/responses. Responses highlighted benefits from lifestyle, socioeconomic, and psychological perspectives. Three major themes emerged: 1. Convenience and reduced burden (e.g., fewer visits, less school/work disruption). 2. Reduced procedural anxiety due to streamlined scheduling. 3. Need for clearer pre-procedure communication among some families. These themes complement the overall positive sentiment observed in the free-text comments. Word cloud depictions of these comments are included in Figure 3. Additionally, 0 (lowest satisfaction) to 5 (highest satisfaction) ratings of the endoscopy experience were gathered from all OAE patients and the mean rating was 4.98.

### 3.5. Points for Consideration When Implementing Open Access Endoscopy

**Figure 4:**
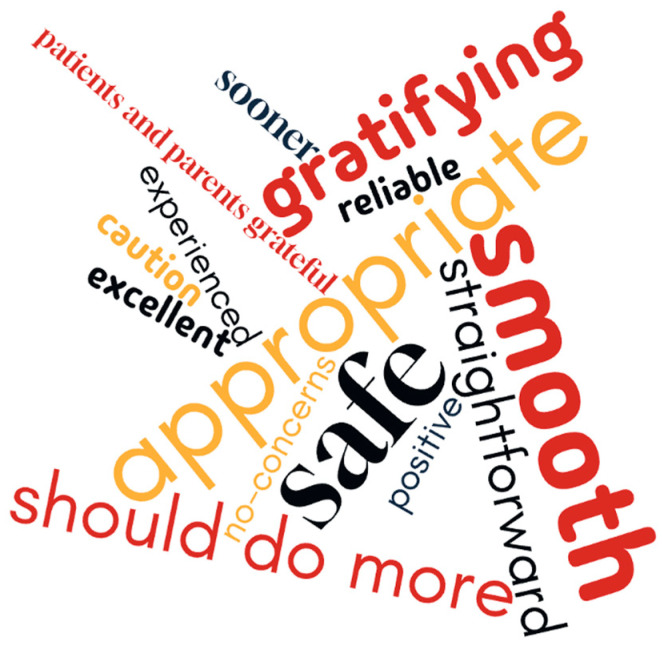
Word cloud depicting endoscopist and scheduling team perceptions of the open access endoscopy experience reported during pre- and post-procedure open-ended survey. Word cloud shown for illustrative purposes; themes derived from qualitative analysis are summarized in the text.

Feedback from endoscopists and scheduling/administrative teams revealed some points for consideration related to the OAE process (Figure 4). These key factors essential for the success of an open access pediatric endoscopy program are delineated in Table 1 and include assessment of the appropriateness of endoscopy referrals and ensuring that parents and patients fully understand the endoscopic plan for informed consent. Additionally, the importance of patient preparedness for the procedure is emphasized, which encompasses aspects such as bowel preparation and management of anti-coagulation. Finally, these points for consideration highlight the need for ensuring the yield of the procedure is adequate through appropriate biopsies and evaluations, as well as the assurance of proper follow-up care with the referring care team.

## 4. Discussion

By allowing direct referrals from primary care providers and outside gastroenterologists, OAE has the potential to streamline the process of obtaining necessary endoscopic evaluations, thereby reducing costs and enhancing overall efficiency in patient care. This is a well-established pathway for endoscopic care in adult patients—one that has stood the test of time and is now considered standard of care for the majority of endoscopic procedures [3,4,5]. Adult OAE programs have consistently demonstrated high diagnostic yield and low adverse event rates, supporting their widespread adoption. Our findings similarly showed no adverse events and successful completion of all procedures; however, unlike the large adult datasets, our study represents only a small, highly selected pediatric cohort. While these preliminary observations parallel adult safety data, definitive conclusions about pediatric safety cannot yet be drawn. Our experience suggests that pediatric OAE may be feasible and appeared safe in this small, carefully selected cohort. However, the absence of adverse events in 54 patients should be interpreted cautiously and does not establish generalizable safety or effectiveness.

The only prior pediatric OAE study [8] focused on patient and caregiver mood and reported lower satisfaction among OAE recipients compared with traditional scheduling. In contrast, our families generally expressed high satisfaction and identified several practical advantages. Unlike the earlier study, our report includes procedural outcomes and safety assessment. These differences highlight how institutional processes, communication strategies, and patient selection may substantially influence satisfaction with pediatric OAE.

One of the significant advantages of implementing an OAE program is the potential to minimize disruptions to patients’ daily lives, particularly in terms of school attendance. By facilitating quicker direct access to endoscopic procedures when appropriate, we can reduce the number of school days missed by children due to medical appointments and procedures. Additionally, the OAE approach may help alleviate medical anxiety for both patients and their families. The streamlined process can foster a sense of empowerment and control, as families are able to navigate the healthcare system more efficiently.

While feedback from patients and endoscopists was overall very positive, feedback from patients, parents, and endoscopists has highlighted some potential points for consideration in an OAE program and these will be valuable for other institutions as they contemplate implementation of OAE programs. These points for consideration include the validity of reliance on pre-procedural assessments from outside referring physicians and care teams, the need for clear communication about the OAE process to referring care teams and patients, and the importance of ensuring that patients are adequately prepared for their procedures. In response to these points for consideration, we reflected on our practices and refined and standardized our communication processes. We regularly conduct interim evaluations of these processes to assess the effectiveness of these refinements. Continuous quality improvement is essential in any healthcare initiative such as OAE, and our commitment to adapting our program based on the input of parent/patient/endoscopist stakeholders is instrumental to the success of this program.

This pilot study has several limitations. The sample size was small, drawn from a single tertiary center, and lacked a comparison group. Patients were screened by experienced endoscopists and had minimal comorbidities, introducing selection bias that likely reduced procedural risk. The qualitative feedback may also be subject to response and social desirability biases. Together, these factors limit the generalizability of our findings and underscore the need for larger, multi-center studies. The implementation of OAE in pediatric gastroenterology is an area that warrants further study and discussion at the level of endoscopy societies. As the landscape of healthcare continues to evolve, it is of vital importance to explore innovative approaches that enhance access to care while reducing costs and maintaining safety and quality. Future research could focus on larger-scale, multi-center studies to evaluate the long-term outcomes of OAE in pediatric populations, including patient satisfaction, cost-effectiveness, incidence of adverse events and effectiveness in management of the symptoms and conditions for which the patients are undergoing treatment. This sort of larger scale study would be important for establishing best practice guidelines for OAE in pediatrics to guide care.

In conclusion, this pilot study describing our experience with the pediatric OAE program underscores its potential to improve access to endoscopic services for children and adolescents. The OAE program we developed is now thriving. By leveraging the expertise of experienced endoscopists and refining our practices based on feedback, we continue to enhance the quality of care provided to our OAE patients. Still, implementation of an OAE program is not without challenges and these could limit the feasibility of implementation at some institutions. Potential limitations to feasibility of implementation include resource availability, availability of experienced endoscopists comfortable performing OAE procedures and established institutional workflows and referral processes. In pediatric settings, OAE adoption must also address several factors not typically encountered in adult practice, including the need for age-appropriate preparation, caregiver coordination, more variable sedation requirements, and the developmental ability of children to participate in preprocedural discussions. These elements may affect both safety and family satisfaction and should be considered when evaluating broader implementation. As we move forward, collaboration and dialog within the pediatric gastroenterology and pediatric endoscopy community will be essential in advancing the implementation of OAE and ensuring that it meets the needs of gastroenterology providers, pediatric patients and their families.

In summary, our early experience indicates that open-access endoscopy may be a feasible approach for selected pediatric patients when performed by experienced endoscopists within structured institutional workflows. While no adverse events were observed, the limited sample size precludes firm conclusions about safety or effectiveness. Broader evaluation across diverse centers and patient populations will be essential to determine the role of OAE in pediatric practice.

## Figures and Tables

**Table 1:** Factors to Consider and Address for a Successful Open Access Pediatric Endoscopy Program.

Appropriateness of endoscopy referral
Adequate parent/patient understanding of endoscopic plan for true informed consent
Patient preparedness for endoscopy (including bowel preparation, anti-coagulation, etc.)
Yield of procedure (including appropriate biopsies/evaluations)
Assurance of appropriate follow-up

## Data Availability

The original contributions presented in this study are included in the article. Further inquiries can be directed to the corresponding author(s).
